# Substance abusers' personality disorders and staff members' emotional reactions

**DOI:** 10.1186/1471-244X-8-21

**Published:** 2008-04-10

**Authors:** Birgitte Thylstrup, Morten Hesse

**Affiliations:** 1 Aarhus University, Centre for Alcohol and Drug Research, Købmagergade 26E, 1150 Copenhagen C, Denmark

## Abstract

**Background:**

Previous research has indicated that aggressive behaviour and DSM-IV cluster B personality disorders (PD) may be associated with professionals' emotional reactions to clients, and that cluster C PD may be associated with positive emotional reactions.

**Methods:**

Staff members recruited from workshops completed a self-report inventory of emotional reactions to patients, the Feeling Word Checklist-58, and substance abusers completed a self-report of DSM-IV personality disorder, the DSM-IV and ICD-10 Personality Disorder Questionnaire. Correlational analysis and multiple regression analysis was used to assess the associations between personality disorders and emotional reations.

**Results:**

Cluster B disorder features were associated with feeling distance to patients, and cluster C disorder features were associated with feeling helpful towards patients. Cluster A disorders had no significant impact on emotional reactions.

**Conclusion:**

The findings confirm clinical experiences that personality disorder features in patients with substance abuse have an impact on staff members reactions to them. These reactions should be considered in supervision of staff, and in treatment models for patients with co-morbid personality disorders and substance abuse.

## Background

The idea that professionals emotional reactions to patients are an important part of psychiatric treatment dates back to the work of Freud, who coined the term countertransference to describe such reactions. Freud observed that the patient's influence on the analyst's unconscious feelings could interfere with treatment. Later observations tend to support the view that emotional reactions are able, not only to interfere with treatment, but also have diagnostic and therapeutic relevance and in many situations, even facilitate rather than interfere with treatment [e.g. [1,2]]. The therapeutic and diagnostic relevance could be information to the therapists about problems the patients are struggling with, secondly it may affect the outcome through the presence, or lack of, empathic attunement [3].

Recently, Sattar and colleagues have pointed out that the term countertransference reaction refer to reactions that occur within the therapeutic alliance, but that many other factors outside the therapeutic alliance can influence staff members' emotional reactions [4]. In this article we use the term "emotional reactions to clients" to refer to all feelings that are evoked in professionals working with patients receiving treatment for psychiatric conditions. The reactions can occur during assessment or treatment, are evoked in dealing with the client, and have the potential to affect treatment.

Only quite recently have researchers begun to study emotional reactions to clients using structured instruments to assess such emotional reactions to patients [5-7]. While there is no universal agreement as to what feelings are important in emotional reactions to patients, both positive feelings and negative feelings are studied.

Researchers conducting factor analyses of emotional reactions to patients have reported a number of factors underlying different instruments. Factor analyses of the Feeling Word Checklist have found 2 superordinate factors (labelled helpfulness and distance) [8,9]. In a large sample, Røssberg and colleagues reported seven factors underlying feelings: important, confident, rejected, on guard, bored, overwhelmed and inadequate [9]. A recent study by Betan and colleagues presented a new instrument, the Countertransference Questionnaire [10]. They reported 8 factors underlying the responses to patients: overwhelmed/disorganized, helpless/inadequate, positive, special/overinvolved, sexualized, disengaged, parental/protective, and critized/mistreated. Most of these scales showed an assocation with a cluster B symptom count, especially overwhelmed/disengaged and criticized/mistreated. One scale, parental/protective, showed an association with cluster C symptoms. However, a limitation of this study is that the same therapists rated both the personality disorders and the counter-transference feelings. Therefore the results could not conclusively show whether the assocation is between patients' characteristics and therapists' emotional reactions, or between therapists' emotional reactions and therapists' perception of patients. Here a study by Hoffart and colleagues with patients with agoraphobia showed that emotional reactions to patients were associated with total PD symptom count [11]. The personality disorders in this group were mainly cluster C disorders, in particular avoidant personality disorder.

Concerning the connection between therapists' emotional reactions and characteristics of the patients and therapists, a study by Holmqvist and Armelius suggested that staff members' personality characteristics are strong predictors of countertransference reactions [12].

Other research has shown that some personality disorders such as antisocial, narcissistic and histrionic personality disorder, are associated with aggressive defense mechanisms or strategies of coping with distress that may cause discomfort in professionals, e.g. "turning against others" [13]. Here Perry and Perry have shown that narcissistic personality disorder is associated with particular types of interpersonal and intrapersonal conflicts in therapy [14]. Some studies have also suggested that patients' level of aggressiveness is quite important in understanding emotional reactions to patients, and that level of suicidality may be associated with both feelings of being important for the patient, and a range of negative feelings [15].

Findings from a study be Betan and colleagues suggested that in particular the dramatic-erratic cluster B personality disorders were associated with negative feelings towards patients, and with the absence of positive feelings [10].

### Aim of the study

The aim of the current study is to analyze how personality disorders are associated with professionels' emotional reactions to patients, when the two are measured by independent sources: self-reported personality disorder features and staff-reported emotional reactions to patients.

## Methods

Subjects were recruited through workshops where participants, addiction counsellors, social workers, nurses or psychologists, learned about personality disorders and the self-report instrument used in this study to assess personality disorder features, the DSM and ICD-10 Personality Questionnaire (DIP-Q). All patients participating were referred by local authorities for substance abuse treatment, and deemed in need of drug abuse treatment. Only patients from treatment units that served only drug abusers were included.

Staff members were instructed to hand out the DIP-Q to patients in their care. They were also requested to inform patients, that the data from the instrument would be used to both research purposes and in their own treatment, and that they would receive a personalized feedback on their test results. On the front of the DIP-Q, we added information about the potential use of the data from the questionnaire for research purposes. The professionals were not asked to include all patients in their units, thus the sample is a convenience sample. There are no institutional review boards in Denmark for research on human subjects that only includes psychosocial assessment or intervention. The research was carried out with respect for the Helsinki declaration.

### Instruments

#### The DSM-IV and ICD-10 Personality Questionnaire

The DSM-IV and ICD-10 Personality Questionnaire [DIP-Q] was used as the measure of personality pathology. The DIP-Q is a self-report questionnaire for screening for DSM-IV and ICD-10 PDs, plus schizotypal disorder in ICD-10 [16]. The instrument is highly similar to other questionnaires measuring PDs, such as the SCID-IIQ, and the PDQ-R. It consists of 151 statements that must be rated as true or false, and three self-rating scales: severity of current events, global assessment of functioning, axis V of the DSM-IV for past year and global assessment of functioning for recent weeks. The DIP-Q was constructed through a consensus process. First, four psychiatrists selected a range of statements considered representative of the diagnostic criteria for each personality disorder. These statements could answered in a true/false format. The representative statements were then reviewed and validated by a second set of independent psychiatrists [17]. A translation and an English version was made available from the Swedish authors. No details of this translation were available and therefore a new Danish translation was made based on the English and Swedish versions, and compared with the original translation.

Studies show indications of concurrent [16] and predictive [18] validity of the instrument. From the DIP-Q, we calculated the number of criteria in each of the three clusters of the DSM-IV, and for each personality disorder.

#### The Feeling Word Checklist-58

We used the Feeling Word Checklist-58 to measure emotional reactions to patients. The Feeling Word Checklist-58 (FWC-58) is based on the FWC developed by Whyte et al. [7] but expanded with 28 items – 23 items were feeling words that experienced therapists found were lacking in the original FWC, and five were taken from the PANAS scale developed by Watson & Lee [19]. The new items were mainly connected to feelings of security, being invaded, idealized and devalued. It was developed in Norwegian, but translated from the English version and back-translated several times by the authors and several English native-speakers.

The instruction to the form is: When I am in conversations with patient ___ I feel ...". Each feeling word is rated on a 5-point likert scale ranging from "Not at all" to "very much". Røssberg and colleagues conducted a factor analysis of the instrument, and derived to superordinate factors and 7 lower-order factors [9]. The two superordinate factors were labelled helpfulness and distance, and the lower-order factors were labelled important, confident, rejected, on guard, bored, overwhelmed and inadequate.

#### Statistical analyses

Power analysis showed that to detect correlations of 0.30 with an alpha of 0.05 and two tails, we needed 82 subjects to obtain a power of 0.80. We decided on 0.30 as a realistic correlation, based on previous studies of countertransferrence [10]. We also conducted power analysis for a regression analysis with 3 predictors. We assumed that only one factor would be independently associated with each of the two dependent variables (see below). If the two covariates explained 1% of the variance, and the third covariate explained 10% of the incremental variance, the number needed to obtain a power of 0.80 was 74 with an alpha of 0.05.

We first conducted two regression analyses entering criteria count for each of the three DSM-IV clusters (A: Odd-eccentric, paranoid, schizoid, schizotypal; B: Dramatic-erratic, antisocial, borderline, histrionic, narcissistic; C: Anxious-fearful, avoidant, dependent, obsessive-compulsive), as predictors and the two main factors of the FWC as dependent variables in each analysis (i.e., helpfulness and distance). Symptom counts of all three clusters were entered simultaneously in the regression analyses. Before conducting these analyses we rank transformed all variables to reduce the impact of violations from normality, as several of the FWC scales had a small number of outliers.

In the next regression analyses, we regressed the higher order factors on all the Cluster diagnoses within any cluster with a significant impact on that factor, again entering all disorders in a cluster in one model. In a final step, we analyzed significant relationships by using the relevant FWC "small" (lower order) scales as dependent variables.

We also report the simple non-parametric Spearman correlations between all scales of the FWC, both the superordinate and the lower-order facets, and all DSM-IV criteria counts.

## Results

The sample were 83% men and 17% women. The patients came from a total of 6 different treatment facilities, and 44% were from drug free inpatient treatment units, that is, inpatient treatment centres that provided longterm inpatient treatment for substance use disorders. The remaining patients were from outpatient units. None of the treatment facilities were addiction-as-disease models of treatment, and the units generally used a mixture of cognitive-behavioural and general social work models in their treatment approach. The mean age was 33.3 years (range: 17–57). All treatment units served only substance abusers.

Prevalence of screen-positive values on the DIP-Q are shown in Table 1. The overall prevalence of personality disorder was 92%, slightly higher than studies using semi-structured interviews [20], but similar to studies using self-report questionnaires such as the Millon Clinical Mulitiaxial Inventory-III [21].

**Table 1:** Prevalence of screen-positive for personality disorder features

Paranoid	66%
Schizoid	13%
Schizotypal	32%
Antisocial	57%
Borderlin	70%
Histrionic	22%
Narcissistic	30%
Avoidant	59%
Dependent	40%
Obsessive-compulsive	30%
At least one	92%
More than one	78%

In the first analyses, we used the number of items for all personality disorders in each of the DSM-IV clusters (A, B and C). The two higher-order factors of the FWC-58 were regressed on the PD cluster scores. The results are summarized in Table 2. All the Spearman rank order correlations are reported as extra material (see Additional file 1).

**Table 2:** Regression analyses results

	**Beta**	**Standard error**	**t(85)**	**p**
**Helpfulness**				
Cluster A	-0.14	0.13	-1.03	0.31
Cluster B	0.00	0.13	0.02	0.98
Cluster C	0.33	0.14	2.41	0.02
Intercept			3.50	0.00
**Distance**				
Cluster A	0.01	0.13	0.08	0.94
Cluster B	0.37	0.13	2.97	0.00
Cluster C	-0.15	0.13	-1.15	0.25
Intercept			3.10	0.00

For helpfulness, the overall proportion of variance accounted for was 0.09 (F(3,85) = 2.90, p = 0.04). Cluster C criteria were significantly associated with helpfulness (beta = 0.33, p = 0.02). For distance, the overall proportion of variance accounted for was 0.11 (F(3,85) = 3.53, p = 0.02). Distance was significantly associated with cluster B criteria (beta = 0.36, p = 0.004).

In the second step, we conducted a regression analysis with helpfulness as the dependent variable, and all cluster C disorders as predictors, to assess which cluster C disorders were responsible for the positive association between cluster C and helpfulness. Of the cluster C disorders, avoidant personality disorder features were significantly independently associated with helpfulness (beta = 0.26, p = 0.04), and the overall model was significant (r^2 ^= 0.06 after controlling for degrees of freedom; p = 0.04). As can be seen in Table 3, avoidant personality disorder features were associated bivariately with both feeling important and confident.

**Table 3:** Spearman Rank Order Correlations between self-reported personality disorder features and staff members' reactions

	**Avoidant personality disorder**
**Helpfulness**	
Important	*0,20
Confident	*0,23

	**Antisocial Personality disorder**

**Distance**	***0,42
Rejected	*0,25
On guard	***0,39
Bored	*0,27
Overwhelmed	***0,39
Inadequate	*0,26

Notes: *** p < 0.001. ** p < 0.01. * p < 0.05. Only correlations between Feeling Word Checklist scales and personality disorder criteria counts that were found to be significant in regression analyses are reported (see methods section).

Further, we conducted a regression analysis with distance as the dependent variable, and all cluster B disorders as predictors, to assess which cluster B disorders were responsible for the positive association between cluster B and distance. The regression model was significant (r^2 ^= 0.16 after adjusting for degrees of freedom, p < 0.01), and antisocial personality disorder features were associated with distance (Beta = 0.47, t(78) = 3.83, p = 0.0003). As can be seen in Table 3, antisocial personality disorder features were associated with practically every factor related to distance, with the strongest correlations with feeling on guard (rho = 0.39) and feeling overwhelmed (rho = 0.39).

## Discussion

This study is the first to show that self-reported personality disorder features are associated with staff members emotional reactions to patients. Studies that have used staff rated personality disorder features run the risk of confounding diagnosis and rating of reactions, a risk that was substantially reduced in this study. Never the less, the results were similar to what has been reported in other studies [10]. The presence of cluster C features induced helpful feelings in staff members, where cluster B disorders, increased feelings of distance. Further analyses suggested that avoidant personality disorder features were responsible for the association between cluster C disorders and helpfulness, but that antisocial personality disorder features were responsible for the association between cluster B disorders and distance.

The sample had a high proportion of men, and the mean age was in the early 30ties. Although the sample was a convenience sample, its age and gender distribution is highly representative of drug abusing patients in Denmark, both outpatient [22,23] and inpatient [24].

All of the patients were substance abusers, and the staff members' general response to substance abusers is not the focus of this study. However, reactions to substance abusers in general is an interesting question, and in studies with both substance abusers and general psychiatric patients, it would be interesting to see, if the presence of substance use problems is associated with reactions to patients, after controlling for the presence or absence of personality disorders.

In terms of the individual personality disorders and specific scales, the findings should be interpreted with some caution, and require replication. However, a clear pattern did emerge: cluster A symptoms had little if any impact on staff emotional reactions. In cluster B, antisocial personality disorder features influenced every feeling related to distance, with the strongest associations with feeling on guard and feeling overwhelmed. The manipulative and at times aggressive behaviours of patients with antisocial personality disorder seem to be a reasonable explanation for this pattern of reactions.

Borderline personality disorder features were somewhat to our surprise not strongly related to staff reactions, and the few reactions that did occur were related to feelings of helpfulness. This runs counter to popular belief that borderline patients are difficult to handle [25]. One explanation is that patients with borderline personality disorder traits also reach out to receive help and support, and therefore may be more open to receive therapeutic support compared with other patients with substance use disorders. Also, patients with borderline personality disorder have a very fluctuating course of illness, and often the impulsive acting-out associated with borderline personality disorder features seems to be quite time-limited, whereas emotional problems persist and call for attention [26].

Cluster C disorder were associated with feelings of helpfulness, and the association was mainly due to correlations between avoidant personality disorder features and helpfullness. Cluster C disorders may represent relatively 'normal' problems that are easily understood by staff members compared with cluster A and B disorders. However, an alternative explanation is that clinicians working with an avoidant patient may compensate for the patient's insecurity and uncertainty by taking a lead in the contact with the patient: taking the lead may produce experience of more certainty and feelings of being more important in the treatment relation.

If personality disorders are systematically linked to the staffs' emotional reactions to the patients, it suggests that clinicians regardless of therapeutic orientation can make diagnosis and therapeutic use of their own response to their patient.

We will here stress that we do not consider feeling distance to patients a negative thing. Reacting with being on guard or feeling rejected by a patient who is manipulating, lying and trying to deceive staff members or other patients is a healthy and normal reaction, and gives the staff member a chance to remain authentic and realistic in the contact with the patient. Also, the fact that the staff member reacts with feelings of helpfulness to the vulnerable patient may be a reasonable reflection of the patient's vulnerability and need for support and comfort.

Some strengths characterize this study. Personality disorder assessment and staff emotional reactions were each measured independently. Therefore, we can be fairly sure that criterion and predictor were not confounded. Further, we used instruments that have been used in a number of previous studies, and the DIP-Q is directly based on the DSM-IV criteria, and has a factor structure that is close to the factor structure of personality disorders in general [27].

There are several limitations to this study that must be acknowledged. First of all, the study used a convenience sample of those patients that staff members chose for axis II screening, and the clinicians were taken from workshops. Findings based on convenience samples such as this should be interpreted with some caution, and replicated with consecutive or true random samples of patients, to ensure both external and internal validity.

Secondly, although our power analyses showed that the number of subjects were acceptable for measuring modest correlations and conducting the regression analyses, several analyses were conducted with the sample, and the findings are in need of replication.

## Conclusion

The findings confirm clinical experiences that personality disorder features in patients with substance abuse have an impact on staff members reactions to them.

## Competing interests

The author(s) declare that they have no competing interests.

## Authors contributions

Both authors planned and discussed the study, and arranged data collection. MH drafted the manuscript and carried out statistical analyses. Both authors revised and discussed the manuscript.

## Pre-publication history

The pre-publication history for this paper can be accessed here:



## Supplementary Material

Additional file 1 Spearman Rank Order Correlations between self-reported personality disorder features and staff members' reactions Click here for file
