# Supplementary material for: Substance abusers' personality disorders and staff members' emotional reactions
Source: BMC Psychiatry. 2008 Apr 10;8:21. doi: 10.1186/1471-244X-8-21 (PMC2322971; doi:10.1186/1471-244X-8-21)
Supplement: Additional file 1 — Spearman Rank Order Correlations between self-reported personality disorder features and staff members' reactions [file 1471-244X-8-21-S1.doc]

# Appendix table. Spearman Rank Order Correlations between self-reported personality disorder features and staff members’ reactions

|  | | PAR | SCD | SCT | CD | ASP | BOR | HIS | NAR | AVO | DEP | COM |
| --- | --- | --- | --- | --- | --- | --- | --- | --- | --- | --- | --- | --- |
| Helpfulness | | 0,06 | -0,02 | -0,02 | 0,06 | 0,03 | *0,23 | 0,06 | 0,04 | ***0,26** | 0,19 | 0,05 |
|  | Important | 0,01 | 0,02 | -0,08 | 0,08 | 0,06 | *0,22 | 0,05 | -0,00 | ***0,20** | *0,23 | 0,03 |
|  | Confident | 0,04 | -0,09 | 0,05 | -0,04 | -0,07 | 0,09 | 0,01 | 0,07 | ***0,23** | -0,04 | 0,07 |
| **Distance** | | 0,15 | 0,00 | 0,13 | ***0,37 | *****0,42** | 0,08 | 0,13 | 0,17 | -0,10 | 0,17 | 0,12 |
|  | Rejected | 0,14 | -0,06 | 0,09 | **0,27 | ***0,25** | 0,02 | 0,06 | 0,05 | -0,02 | 0,16 | 0,09 |
|  | On guard | 0,08 | -0,01 | 0,11 | **0,29 | *****0,39** | 0,09 | 0,12 | 0,14 | -0,07 | 0,17 | 0,08 |
|  | Bored | 0,17 | 0,02 | 0,11 | *0,25 | ***0,27** | 0,07 | 0,16 | 0,05 | -0,03 | 0,18 | 0,12 |
|  | Overwhelmed | 0,07 | -0,08 | 0,14 | **0,34 | *****0,39** | 0,10 | *0,23 | *0,23 | -0,12 | 0,16 | 0,11 |
|  | Inadequate | 0,11 | 0,12 | 0,07 | 0,17 | ***0,26** | 0,07 | 0,02 | 0,07 | 0,03 | 0,14 | 0,06 |

Notes: *** p<0.001. ** p<0.01. * p<0.05. PAR: paranoid, SCD: Schizoid, SCT: Schizotypal, CD: Conduct disorder, ASP: Antisocial, BOR: Borderline, HIS: Histrionic, NAR: Narcissistic, AVO: Avoidant, DEP: dependent, COM: Obsessive-Compulsive. Correlations in **boldface** are relevant for the present work.
